# Physical activity in relation to risk of chronic obstructive pulmonary disease among Chinese adults: an 11-year prospective study

**DOI:** 10.3389/fspor.2025.1612278

**Published:** 2026-01-09

**Authors:** Hao Wang, Huaidong Du, Lingli Chen, Kaixu Xie, Yuan Cao, Zhengjie Shen, Jun Lv, Canqing Yu, Dianjianyi Sun, Pei Pei, Jieming Zhong, Min Yu

**Affiliations:** 1 Department of Non-Communicable Diseases Control and Prevention, Zhejiang Provincial Center for Disease Control and Prevention, Hangzhou, Zhejiang, China; 2 Clinical Trial Service Unit & Epidemiological Studies Unit (CTSU), Nuffield Department of Population Health, University of Oxford, Oxford, United Kingdom; 3 Department of Non-Communicable Diseases Control and Prevention, Tongxiang City Center for Disease Control and Prevention, Jiaxing, China; 4 Department of Epidemiology & Biostatistics, School of Public Health, Peking University, Beijing, China; 5 Peking University Center for Public Health and Epidemic Preparedness and Response, Beijing, China; 6 Key Laboratory of Epidemiology of Major Diseases (Peking University), Ministry of Education, Beijing, China

**Keywords:** adults, chronic obstructive pulmonary disease, metabolic equivalent task, physical activity, prospective study

## Abstract

**Background:**

Chronic obstructive pulmonary disease (COPD) is a leading cause of chronic morbidity and mortality worldwide. Despite its significant public health burden, few studies have reported on the association of physical activity with incident COPD. This study aimed to determine the association between physical activity and the risk of incident COPD.

**Methods:**

This prospective cohort study analyzed data from 49,482 participants aged 30–79 years enrolled in the China Kadoorie Biobank study in Tongxiang. Baseline assessments were carried out between August 2004 and January 2008. Physical activity was measured using an interviewer-administered questionnaire and quantified as metabolic equivalent of task hours per day (MET-h/day). Participants were categorized into quartile-based activity groups for analyses. All participants were followed up from the date of baseline survey until the date of COPD diagnosis, death, loss to follow-up, or 31 December 2017, whichever occurred first. Incident COPD events were obtained periodically through linkage with national insurance electronic systems and death registries. Cox proportional hazards regression was employed to estimate adjusted hazard ratios of COPD in relation to physical activity.

**Results:**

The average of the physical activity level of participants was 31.1 ± 15.1 MET-hours/day. During 551,266 person-years (median 11.5 years) of follow-up, 1,470 incident COPD cases (744 men and 726 women) were documented. After adjusting for socio-demographic status, lifestyle factors (cigarette and alcohol consumption, secondhand smoke exposure, meat and fresh fruit consumption, sleep duration), BMI, and household cooking fuel type, participants with physical activity levels in the highest vs. lowest quartile exhibited a 30% reduced risk of incident COPD (HR = 0.70, 95%CI, 0.54–0.91) in smoking men. However, no significant association was observed in women (HR = 0.99, 95%CI, 0.77–1.27) or non-smoking men (HR = 1.05, 95%CI, 0.41–2.46).

**Conclusion:**

Physical activity is inversely associated with incident COPD risk in smoking men but not in women or non-smoking men.

## 1 Introduction

Chronic obstructive pulmonary disease (COPD) remains a leading cause of chronic morbidity and mortality worldwide. Between 1990 and 2019, estimated incident COPD cases surged by 86% (from 8.72 million to 16.21 million), while COPD deaths increased by 30% (from 2.52 million to 3.28 million) (1). In China, COPD accounted for 1.04 million deaths in 2019, representing approximately one-third of global COPD deaths, and the number of patients with COPD was estimated to be 45.16 million (2). One nationally representative study of 50,991 adults aged 20 years or older from ten provinces in mainland China, conducted between 2012 and 2015, indicated that the prevalence of spirometry-defined COPD was 8.6%, accounting for 99.9 million people with COPD in China (3). At the same period, another nationally representative study of 66,752 adults aged 40 years or older from 125 counties/districts in mainland China suggested the prevalence of COPD was 13.6% (4).

Smoking is considered to be the most important risk factor for COPD, and 51% of COPD deaths in the Chinese population in 2019 were estimated to be caused by smoking (2). Smoking patterns in China differ from those typically seen in Western populations, including China's high prevalence of cigarette smoking among men (50.8%) but low prevalence among women (2%) (5), a low prevalence of smoking cessation (15%) (5), and a low frequency of e-cigarette use (1.6%) (6).

Physical activity encompasses four domains: occupational, household, commuting, and leisure-time physical activity. More recent research on physical activity has predominantly focused on its associations with various health conditions, including cancers (7–9), cardiovascular diseases (9–11), metabolic disorders such as obesity (12–14) and diabetes (14–16), dementia (17, 18), chronic kidney disease (19), and insomnia symptoms (20). Furthermore, most studies examining the association of physical activity with COPD have concentrated on patients with existing COPD (21, 22)—some suggesting that physical activity may serve as the strongest predictor of all-cause mortality in patients with COPD (22). Little is known about the relevance of physical activity for COPD risk among the general population. Tongxiang is located on the eastern coast of China, with a typical subtropical monsoon climate and an average annual precipitation of more than 1,200 mm. The terrain is mainly flat, with a dense network of rivers. Hence, this study aims to determine the association between physical activity and risk of incident COPD using data from Tongxiang Zhejiang region within the China Kadoorie Biobank.

## 2 Materials and methods

### 2.1 Study population and design

CKB study is an ongoing, nationwide, prospective cohort study. Details of the CKB study design and method have been reported previously (23–25). The data utilized in the current study were derived from Tongxiang, one of the 10 study regions in China. Briefly, 57,704 eligible participants aged 30–79 years old participated in the baseline survey between August 2004 and January 2008.

### 2.2 Assessment of physical activity

The Detailed methodology for assessing physical activity in this study, including the specific questionnaire items employed, has been previously documented (12, 26). At baseline, participants completed a physical activity questionnaire that captured four domains: occupational, household, commuting, and leisure-time physical activity. For each domain, the questionnaire assessed three key dimensions: frequency, intensity, and duration of participation.

To quantify the amount of physical activity, metabolic equivalent tasks (METs) based on the 2011 update of a comprehensive compendium of physical activities were utilized (27). The MET value for a given activity represents the ratio of energy expended per kilogram of body weight during that activity to that expended while sitting quietly (Supplementary Table S1). The MET of each activity was multiplied by its frequency and duration to calculate total physical activity in MET hours per day (MET-h/d). Total physical activity was calculated by summing the MET-hours of the four physical activity domains.

### 2.3 Assessment of covariates

Detailed information on covariates was collected by trained staff at baseline using a standardized questionnaire. The collected information encompassed four domains: socio-demographic characteristics (age, sex, education level, marital status, and household annual income), lifestyle factors (cigarette and alcohol consumption, secondhand smoke exposure, intakes of fresh fruit and meat, and sleep duration), personal medical history (chronic bronchitis/emphysema, asthma, tuberculosis, cancer, stroke, diabetes, and ischemic heart disease), and household air pollution (i.e., cooking fuel type and heating fuel type).

Anthropometric measurements were measured using calibrated instruments by trained staff. Standing height was measured without shoes to the nearest 0.1 cm using a stadiometer. Body weight was measured to the nearest 0.1 kg with a body composition analyzer (TANITA-TBF-300GS, Tanita Corp., Tokyo, Japan), subtracting the weight of clothing according to season (0.5 kg in summer and 2.0–2.5 kg in winter). Body mass index (BMI) was calculated as weight in kilograms divided by the square of height in meters. Overweight was defined as 24.0 ≤ BMI < 28.0 kg/m^2^, and obesity was defined as BMI ≥ 28.0 kg/m^2^ (28).

A non-fasting venous blood sample was obtained for immediate on-site testing of plasma glucose level. Random plasma glucose level was measured using the SureStep Plus System (Johnson & Johnson, New Brunswick, NJ, USA). Participants without self-reported diabetes but with a random plasma glucose level ≥11.1 mmol/L or fasting plasma glucose level ≥7.0 mmol/L were defined as having screen-detected diabetes.

Pre-bronchodilator FEV1 and FVC were evaluated using a handheld Micro Spirometer (MS01, CareFusion UK Ltd, Basingstoke, UK) by trained technicians following recommended procedures (29). After performing practice exhalations, participants completed two successful maneuvers, as judged by the technician, with the results recorded for analyses.

In the present study, participants with a self-reported history of physician-diagnosed chronic disease (1,436 with chronic bronchitis or emphysema, 288 with asthma, 333 with tuberculosis, 163 with cancers, 349 with strokes, 464 with heart disease, and 1,380 with diabetes) and 1,432 participants with screen-detected diabetes at baseline were excluded. Additionally, 2,377 participants who had airflow obstruction defined as the ratio of forced expiratory volume in 1 s to forced vital capacity (FEV1/FVC) < 0.7 were excluded. After these exclusions, a total of 49,482 (20,172 men, 29,310 women) participants remained for inclusion in the final analysis (Supplementary Figure S1).

### 2.4 Follow-up for incident COPD

All participants were followed up from the date of baseline survey until the date of COPD diagnosis, death, loss to follow-up, or 31 December 2017, whichever came first. Incident COPD events were mainly obtained via the national health insurance electronic systems, which were linked to hospitalization records provided by certified physicians. These systems covered more than 98% study participants in Tongxiang. COPD events were also acquired periodically through linkage with death registries. In addition, to ascertain hospitalization episodes, CDC staff actively followed up with individuals who were not registered in the health insurance electronic system. Trained staff, blinded to the baseline information, coded all cases with the 10th revision of the International Classification of Diseases (ICD-10). For the present analysis, we included COPD cases as J41–J44. Despite spirometry's underutilization in China, COPD diagnoses recorded in CKB—whether based on spirometry or clinical assessment—were found to be highly reliable. To evaluate the validity of diagnosis, 1,069 randomly selected COPD cases in the CKB study were adjudicated by five experienced physicians, and the results illustrated that the positive predictive value of COPD diagnosis was 85% (30).

### 2.5 Statistical analysis

Physical activity (MET-h/d) was divided into quartiles: quartile 1 < 18.58; 18.58 ≤ quartile 2 < 29.99; 29.99 ≤ quartile 3 < 41.78; 41.78 ≤ quartile 4. Percentage and mean values of characteristics at baseline were calculated according to physical activity quartiles, with adjustment for age and sex as appropriate, using either multiple linear regression (for continuous variables) or logistic regression (for categorical variables) for statistical testing. Analysis of variance or Kruskal–Wallis rank test was used for continuous variables. Cox proportional hazards regression was used to estimate adjusted hazard ratios (HRs) of COPD in relation to physical activity. In Model 1, HRs were adjusted for age at baseline and sex. In Model 2, HRs were further adjusted for education level (no formal education, primary school, middle school, and high school or above), household income (<19,999 CNY, 20,000–34,999 CNY, and ≥35,000 CNY), marital status (married, unmarried), cigarette consumption (never, occasional, former, and current regular), alcohol consumption (never, occasional, former, and current regular), secondhand smoke exposure (<1 time/week, 1–2 days/week, 3–5 days/week, 6–7 days/week), meat and fresh fruit consumption (daily, non-daily), sleep duration (continuous), and BMI (continuous). In Model 3, HRs were further adjusted for household cooking fuel type (no cooking, coal/wood/charcoal, clean fuel). Considering 99.4% participants did not use household heating fuel in winter (31), heating fuel type was not adjusted for in our study. Subgroup analyses examined the associations in subgroups defined by baseline age (30–49 years, 50–79 years), sex (men, women), education level (no formal education, primary school or above), household annual income (<35,000 CNY, ≥35,000 CNY), smoking status (non-smokers, smokers), and alcohol status (non-drinkers, drinkers), meat consumption (daily, non-daily), fruit consumption (daily, non-daily), BMI (<24 kg/m^2^, ≥24 kg/m^2^), secondhand smoke exposure (<1 time/week, ≥1 day/week), cooking fuel type (clean fuel, solid fuel), sleep duration (<10 h/day, ≥10 h/day). Non-smokers were defined as never-smokers. Occasional, former, and current regular smokers were combined into one group (i.e., smokers). Non-drinkers were defined as never-drinkers. Occasional, former, and current regular drinkers were combined into one group (i.e., drinkers). In subgroup analyses, hazard ratios for incident COPD associated with physical activity levels in the highest vs. lowest quartile were calculated. In sensitivity analyses, the first 1 year of follow-up and participants with poor self-rated general health were excluded, separately to reduce the effect of reverse causality, and sedentary leisure time and occupation were further adjusted for in order to reduce the influence of possible residual confounding. We used restricted cubic splines (RCSs) to test for linearity, and RCS was used for physical activity in multivariable-adjusted Cox regression analyses (model 3) for smoking men. All statistical analyses were performed using SAS 9.4 (SAS Inst., Cary, NC, USA) and R (version 4.1.1; R Foundation for Statistical Computing) with 2-sided tests with a significance level of 0.05.

## 3 Results

### 3.1 Characteristics of participants

Among the 49,482 participants included, the mean (SD) baseline age was 51.7 (9.5) years, and 59.2% were women. Overall, 38.2% of participants had a household income more than 35,000 CNY, 27.8% of participants were current smokers, 16.9% were current drinkers, and 17.2% were exposed to secondhand smoke ≥6 days per week. The proportion of participants who use clean fuel for cooking was 31.6% (Table 1). As compared with participants with low physical activity, participants with high physical activity were more likely to be younger, to be married, to be current smokers or drinkers, to consume meat and fruit infrequently, to have a lower BMI, and to have less sleep duration. They were also less likely to be women, to be well-educated, to have higher household income, to be exposed to secondhand smoke, and to cook with clean fuel.

**Table 1:** Participant characteristics by quartiles of physical activity.

Characteristics	Total (*n* = 49,482) Mean ± SD/percentage	Mean ± SD/percentage				*P* value for trend
		Quartile 1	Quartile 2	Quartile 3	Quartile 4	
		(*n* = 11,683)	(*n* = 12,373)	(*n* = 12,609)	(*n* = 12,817)	
Mean age (years)	51.7 ± 9.5	56.1 ± 10.4	53.1 ± 9.4	50.4 ± 8.7	47.9 ± 7.7	<0.001
Women (%)	59.2	59.0	64.0	53.2	56.7	<0.001
High school education or above (%)	4.0	7.7	4.6	3.0	2.2	<0.001
Married (%)	93.7	92.8	93.6	94.7	95.2	<0.001
Household income ≥35,000 CNY (%)	38.2	45.0	39.1	36.7	36.0	<0.001
Current smokers (%)	27.8	27.3	27.4	28.0	28.3	0.02
Current drinkers (%)	16.9	16.1	17.1	17.2	17.9	<0.001
Secondhand smoking ≥6 days/week (%)	17.2	21.4	17.4	15.3	16.2	<0.001
Consuming meat daily (%)	15.5	19.6	15.7	14.6	14.2	<0.001
Consuming fruit daily (%)	6.8	12.0	7.3	5.2	4.5	<0.001
BMI (kg/m^2^)	22.9 ± 3.1	23.3 ± 3.2	23.0 ± 3.1	22.9 ± 3.0	22.9 ± 2.9	0.004
Sleep duration (hours)	7.6 ± 1.2	7.8 ± 1.3	7.7 ± 1.2	7.6 ± 1.1	7.5 ± 1.1	<0.001
Cooking with clean fuel (%)	31.6	38.9	31.6	27.9	29.8	<0.001

Values are adjusted for age and sex, as appropriate.

BMI, body mass index; SD, standard deviation.

### 3.2 Characteristics of physical activity

The average physical activity (MET-h/d) of participants was 31.1 ± 15.1. Men (31.7 ± 15.3 MET-h/d) had higher physical activity than women (30.7 ± 15.0) (*P* < 0.001). Physical activity (MET-h/d) for participants aged 30–49 years, 50–59 years, and 60–79 years was 35.5 ± 14.8, 30.4 ± 14.3, and 22.7 ± 13.4, respectively (*P* < 0.001) (Table 2).

**Table 2:** Description of physical activity by age groups.

Age groups (years)	Total Mean ± SD	Men Mean ± SD	Women Mean ± SD
30–49	35.5 ± 14.8	34.5 ± 14.8	36.1 ± 14.8
50–59	30.4 ± 14.3	33.3 ± 15.1	28.4 ± 13.5
60–79	22.7 ± 13.4	23.9 ± 14.0	21.7 ± 12.8

### 3.3 Association of physical activity with incident COPD

During 551,266 person-years (median 11.5 years) of follow-up, 1,470 incident COPD cases were documented, including 744 men and 726 women. After adjusting for socio-demographic status, cigarette consumption, alcohol consumption, secondhand smoke exposure, meat and fruit consumption, sleep duration, BMI, and household cooking fuel type, physical activity was inversely associated with the risk of incident COPD among men (*P* for trend = 0.02). In comparison with participants with physical activity levels in the bottom quartile, the adjusted HRs for quartile 2, quartile 3, and quartile 4 among men were 0.96 (95%CI, 0.80–1.15), 0.87 (95%CI, 0.71–1.07), and 0.74 (95%CI, 0.57–0.94), respectively. However, there was no clear association of physical activity with risk of incident COPD among women, with corresponding hazard ratios of 1.22 (95%CI, 1.00–1.47), 1.02 (95%CI, 0.81–1.26), and 0.99 (95%CI, 0.77–1.27), respectively (*P* for trend = 0.78) (Table 3).

**Table 3:** Association of physical activity with risk of incident chronic obstructive pulmonary disease.

Quartiles of physical activity	Case, *n*	Incidence rate (per 1,000 person-years), ‰	Model 1	Model 2	Model 3
			HR (95%CI)	HR (95%CI)	HR (95%CI)
Total					
Quartile 1	511	4.0	1	1	1
Quartile 2	450	3.3	1.16 (1.02–1.32)	1.09 (0.96–1.24)	1.07 (0.94–1.23)
Quartile 3	302	2.1	1.01 (0.87–1.17)	0.95 (0.81–1.10)	0.93 (0.80–1.09)
Quartile 4	207	1.4	0.94 (0.79–1.12)	0.86 (0.72–1.03)	0.85 (0.72–1.02)
*P* value for trend			0.49	0.07	0.05
Men					
Quartile 1	258	5.2	1	1	1
Quartile 2	223	4.6	1.04 (0.86–1.24)	0.97 (0.80–1.16)	0.96 (0.80–1.15)
Quartile 3	163	2.5	0.93 (0.76–1.14)	0.87 (0.71–1.07)	0.87 (0.71–1.07)
Quartile 4	100	1.7	0.81 (0.63–1.03)	0.74 (0.58–0.95)	0.74 (0.57–0.94)
*P* value for trend			0.09	0.02	0.02
Women					
Quartile 1	253	3.2	1	1	1
Quartile 2	227	2.6	1.31 (1.09–1.58)	1.24 (1.03–1.50)	1.22 (1.00–1.47)
Quartile 3	139	1.8	1.11 (0.89–1.38)	1.03 (0.83–1.28)	1.02 (0.81–1.26)
Quartile 4	107	1.2	1.11 (0.87–1.42)	1.00 (0.78–1.29)	0.99 (0.77–1.27)
*P* value for trend			0.46	0.88	0.78

Model 1: adjusted for age and sex.

Model 2: adjusted for age, sex, education level, household income, marital status, smoking status, secondhand smoke exposure, alcohol status, meat consumption, fresh fruit consumption, sleep duration, and body mass index.

Model 3: adjusted for age, sex, education level, household income, marital status, smoking status, secondhand smoke exposure, alcohol status, meat consumption, fresh fruit consumption, sleep duration, body mass index, and household cooking fuel type.

After adjusting for socio-demographic status, behavioral lifestyle, BMI, and household cooking fuel type, in comparison with participants with physical activity levels in the bottom quartile, the adjusted HRs for quartile 2, quartile 3, and quartile 4 among smoking men were 0.90 (95%CI, 0.75–1.09), 0.82 (95%CI, 0.66–1.01), and 0.70 (95%CI, 0.54–0.91), respectively (*P* for trend = 0.005). However, there was no clear association of physical activity with risk of incident COPD among non-smoking men, with corresponding hazard ratios of 1.62 (95%CI, 0.87–3.04), 1.54 (95%CI, 0.76–3.08), and 1.05 (95%CI, 0.41–2.46), respectively (*P* for trend = 0.62) (Table 4).

**Table 4:** Association of physical activity with risk of incident chronic obstructive pulmonary disease by smoking status among men.

Quartiles of physical activity	Case, *n*	Incidence rate (per 1,000 person-years), ‰	Model 1	Model 2	Model 3
			HR (95%CI)	HR (95%CI)	HR (95%CI)
Smokers					
Quartile 1	237	5.4	1	1	1
Quartile 2	201	4.6	0.96 (0.79–1.16)	0.91 (0.75–1.10)	0.90 (0.75–1.09)
Quartile 3	147	2.5	0.87 (0.70–1.07)	0.82 (0.66–1.02)	0.82 (0.66–1.01)
Quartile 4	92	1.7	0.76 (0.59–0.98)	0.71 (0.55–0.91)	0.70 (0.54–0.91)
*P* value for trend			0.03	0.006	0.005
Non-smokers					
Quartile 1	21	3.6	1	1	1
Quartile 2	22	4.8	1.89 (1.03–3.48)	1.70 (0.91–3.19)	1.62 (0.87–3.04)
Quartile 3	16	3.1	1.67 (0.84–3.27)	1.61 (0.79–3.22)	1.54 (0.76–3.08)
Quartile 4	8	1.9	1.26 (0.51–2.89)	1.06 (0.41–2.48)	1.05 (0.41–2.46)
*P* value for trend			0.33	0.59	0.62

Model 1: adjusted for age.

Model 2: adjusted for age, education level, household income, marital status, alcohol status, secondhand smoke exposure, meat consumption, fresh fruit consumption, sleep duration, and body mass index.

Model 3: adjusted for age, education level, household income, marital status, alcohol status, secondhand smoke exposure, meat consumption, fresh fruit consumption, sleep duration, body mass index, and household cooking fuel type.

To further explore the association between METs and the hazards of COPD, we performed sex-specific RCS analyses (model 3). The RCS model did not reach the significance level among smoking men (P for nonlinear = 0.57) (Supplementary Figure S2).

### 3.4 Subgroup analyses

In subgroup analyses, there was an inverse association of physical activity with incident COPD among smokers (HR = 0.73, 95%CI, 0.56–0.95), but no clear association among non-smokers (OR = 1.12, 95%CI, 0.86–1.45) (*P* for heterogeneity = 0.02). The association of physical activity with incident COPD was largely consistent across other subgroups examined, defined by age (P for heterogeneity = 0.34), education level (P for heterogeneity = 0.39), household income (*P* for heterogeneity = 0.11), alcohol drinking (*P* for heterogeneity = 0.77), meat consumption (*P* for heterogeneity = 0.38), fruit consumption (*P* for heterogeneity = 0.30), BMI (*P* for heterogeneity = 0.053), sleep duration (*P* for heterogeneity = 0.43), and cooking fuel type (*P* for heterogeneity = 0.62) (Table 5).

**Table 5:** Adjusted hazard ratios for incident chronic obstructive pulmonary disease associated with physical activity levels in the highest vs. lowest quartile by participant characteristics.

Characteristics	Case, *n*	HR (95%CI)	*P* value for heterogeneity
Age (years)			0.34
30–49	77	0.70 (0.44–1.14)	
50–79	641	0.90 (0.73–1.10)	
Sex			0.004
Men	358	0.73 (0.56–0.93)	
Women	360	1.18 (0.91–1.51)	
Education level			0.39
No formal education	476	0.87 (0.68–1.09)	
Primary or above	242	1.01 (0.76–1.33)	
Household income (CNY)			0.11
<35,000	496	0.99 (0.80–1.23)	
≥35,000	222	0.74 (0.54–1.01)	
Smoking status			0.02
Smokers	359	0.73 (0.56–0.95)	
Non-smokers	359	1.12 (0.86–1.45)	
Alcohol status			0.77
Drinkers	241	0.93 (0.70–1.24)	
Non-drinkers	477	0.88 (0.70–1.11)	
Meat consumption			0.38
Daily	89	1.08 (0.69–1.66)	
Non-daily	629	0.88 (0.71–1.07)	
Fruit consumption			0.30
Daily	35	1.32 (0.60–2.70)	
Non-daily	683	0.89 (0.73–1.08)	
Body mass index (kg/m^2^)			0.053
<24	520	0.83 (0.66–1.02)	
≥24	198	1.17 (0.86–1.60)	
Secondhand smoke exposure			0.64
<1 time/week	422	0.88 (0.68–1.12)	
≥1 day/week	296	0.95 (0.73–1.22)	
Cooking fuel type			0.62
Clean fuel	359	0.95 (0.75–1.21)	
Solid fuel	359	0.87 (0.67–1.14)	
Sleep duration (hours/day)			0.43
<10	658	0.90 (0.74–1.09)	
≥10	60	1.16 (0.60–2.10)	

Hazard ratios: adjusted for age, sex, education level, household income, marital status, smoking status, secondhand smoke exposure, alcohol status, meat and fresh fruits consumption, sleep duration, BMI and household cooking fuel type.

### 3.5 Sensitivity analyses

The association of physical activity with incident COPD did not change appreciably after exclusion of the first 1 year of follow-up or, separately, after exclusion of participants with poor self-rated general health at recruitment. Additional adjustment for sedentary leisure time and occupation had little effect on observed risk estimates (Supplementary Tables S2 and S3).

## 4 Discussion

To the best of our knowledge, this paper is the first to evaluate the association of physical activity with incident COPD with a large prospective design among general populations in Asia. In this large prospective cohort study in Tongxiang Zhejiang region, China, there was a clear inverse association between physical activity levels in smoking men and the risk of incident COPD. In contrast, among women and non-smoking men, there were no clear associations.

### 4.1 Characteristics of physical activity

The average level of physical activity in the current study was 31.1 ± 15.1 MET-h/d, higher than the other 9 CKB study regions, reflecting geographic diversity in physical activity levels in China (32). Consistent with the previous studies (32), men had higher physical activity than women. Similar to previous research (10), participants with high physical activity were more likely to be younger, to be current drinkers, and to have a lower BMI.

### 4.2 Association of physical activity with incident COPD

The National Institutes of Health (NIH)-American Association of Retired Persons (AARP) Diet and Health Study (33), including 113,279 US participants aged 50–70 years, indicated that after adjustment for age, sex, marital status, educational attainment, ethnicity, smoking status, alcohol intake, history of diabetes, height and BMI, when compared with participants without vigorous physical activity, the relative risk (95%CI) for incident COPD among participants who engaged in vigorous physical activity <1 time per week, 1–2 times per week, 3–4 times per week, and ≥5 times per week were 0.85 (0.76–0.95), 0.80 (0.72–0.89), 0.73 (0.65–0.80), and 0.68 (0.61–0.77), respectively. However, NIH-AARP Diet and Health Study failed to further ascertain this association among both men and women. In the present study, a null association of physical activity with incident COPD was observed in women.

It was noteworthy that the protective effect of physical activity on incident COPD was observed only among smokers in the current study, similar to the results of NIH-AARP Diet and Health Study (33). The latter documented that an inverse association of vigorous physical activity with COPD was observed among current smokers, but not among never-smokers. Compared with current smokers without vigorous physical activity, the relative risk (95%CI) for incident COPD among current smokers who engaged in vigorous physical activity ≥5 times per week was 0.65 (0.53–0.80). A study of 12,283 adults aged 45–74 years from the European Prospective Investigation into Cancer-Norfolk (EPIC-Norfolk) Study indicated that the annual decrease change of FEV1 among current smokers was significantly higher than that among never-smokers, and physical activity was associated with higher FEV1 (34). These findings from our study might highlight the importance of enhancing physical activity among smokers. Notably, women in the second quartile had a higher risk of COPD than the reference group in sensitivity analyses. One possible explanation was occupational-related physical activity. One study demonstrated that transportation-related physical activity and recreational physical activity were associated with a lower COPD prevalence, whereas occupational-related physical activity was associated with a higher COPD prevalence (35).

Our study findings have several paramount public health implications. Firstly, these findings of an inverse association between physical activity and incident COPD provide new evidence of the importance of physical activity for the general population. Engaging in physical activity might not only reduce the risk of well-established cancers and cardiovascular diseases, but also of COPD. Hence, active physical exercise should be further advocated among the general population. The China Chronic Disease and Risk Factor Surveillance 2018 of more than 183,000 adults aged ≥18 years, an updated nationally representative study conducted in 298 districts/counties, documented that the overall prevalence of insufficient physical activity was 22.3% (36), indicating insufficient physical activity remained prevalent among the Chinese population. Second, an inverse association was observed only among smokers in the current study, signifying that smokers might experience beneficial effects from engaging in physical activity. Hence, more physical activity should be incorporated into the context of health education targeted at smokers.

Several possible mechanisms could explain this association of high physical activity with incident COPD risk. First, physical activity may have a protective effect against chronic inflammation (37), induced by smoking and exposure to noxious particles and gases. Second, physical activity might contribute to processes of lung repair (38). Third, engaging in physical activity may result in weight loss and body composition change (39), and abdominal obesity has been recognized as a potential risk factor for COPD (33, 40, 41).

It should be noted that the generalizability of the current findings of a null association of physical activity with risk of incident COPD in women should be interpreted with caution. Only 0.9% of women in our study region were current smokers (42), lower than global average (8.5%) (43). In addition, the percentage of solid heating fuel use in winter, as an important source for indoor air pollution, varied by regions in CKB study, higher in Northern regions (e.g., 95.1% in Gansu and 82.1% in Henan) than Southern regions (e.g., 6.4% in Sichuan and none in Zhejiang) (31). A previous meta-analysis indicated that a positive association was observed between use of indoor solid fuels and COPD (44). Hence, the association of physical activity with COPD needs to be further validated, with large-scale prospective studies, in regions with different patterns of smoking and air pollution.

There are several limitations of the present study. First, information on physical activity was self-reported by participants and not objectively measured, which might increase the possibility of measurement error. Second, due to limited participants, we failed to observe a significant association of physical activity with COPD among non-smoking men. Third, the association between domain-specific physical activity (i.e., occupational, household, commuting, and leisure-time physical activity) and COPD was not further ascertained owing to the limited sample size. Fourth, the data was derived from one rural region in the present study, and the results should be interpreted cautiously when generalizing to other populations. Fifth, although several established and potential risk factors for incident COPD were adjusted for in different models, residual confounding by other unmeasured or unknown biological and social factors was still possible.

## 5 Conclusions

In conclusion, the current study presents that physical activity is inversely associated with risk of incident COPD in smoking men, but not in women and non-smoking men.

## Data Availability

The datasets presented in this study can be found in online repositories. The names of the repository/repositories and accession number(s) can be found below: Details of how to access China Kadoorie Biobank data and details of the data release schedule are available from https://ckbiobank.pku.edu.cn/index.htm.
