# Supplementary material for: Physical activity in relation to risk of chronic obstructive pulmonary disease among Chinese adults: an 11-year prospective study
Source: Front Sports Act Living. 2026 Jan 9;7:1612278. doi: 10.3389/fspor.2025.1612278 (PMC12827776; doi:10.3389/fspor.2025.1612278)
Supplement: Supplementary file 1 [file Datasheet1.docx]

Table S1. Physical activity types, MET values, codes and intensity categories

| Activity type | Intensity | MET |
| --- | --- | --- |
| Heavy manual work | Vigorous | 6.5 |
| Manual work | Moderate | 4.5 |
| Standing work | Moderate | 3.8 |
| Sedentary work | Low | 1.8 |
| Manual work in the farming season | Vigorous | 6.3 |
| Semi-mechanized work in the farming season | Moderate | 3.4 |
| Fully mechanized work in the farming season | Low | 2.4 |
| Work outside the farming season | Low | 2 |
| Walking | Moderate | 4 |
| Bicycle | Vigorous | 6.8 |
| Motorbike | Moderate | 3.5 |
| Private or public transportation (such as bus, car, underground, and ferry) | Low | 1.7 |
| Household activity | Low | 2.8 |
| Tai-Chi/qigong/leisure walking | Moderate | 3.3 |
| Jogging/aerobic exercise | Vigorous | 7.4 |
| Ball games | Moderate | 5.5 |
| Brisk walking/gymnastics/folk dancing | Moderate | 4.2 |
| Swimming | Vigorous | 7.2 |
| Other exercise, e.g. mountain walking, home exercise and rope jumping | Moderate | 5.9 |

57,704 eligible participants aged 30-79 years old participated in the baseline survey between 2004 and 2008.

49,482 were followed up from the date of baseline survey until the date of COPD diagnosis, death, or 31 December 2017

1470 incident COPD were documented.

Participants with self-reported history of physician-diagnosed chronic disease were excluded:

Chronic bronchitis or emphysema (n=1436)

Asthma (n=288)

Tuberculosis (n=333)

Cancers (n=163)

Strokes (n=349)

Heart disease (n=464)

Diabetes (n=1380)

Participants with screen-detected diabetes at baseline were excluded (n=1432)

Participants with screen-detected airflow obstruction at baseline were excluded (n= 2377)

Through linkage with death registries and with national health insurance electronic systems.

Through active follow-up annually.

Figure S1. Study flow diagram of cohort selection, inclusion, exclusion, and follow-up


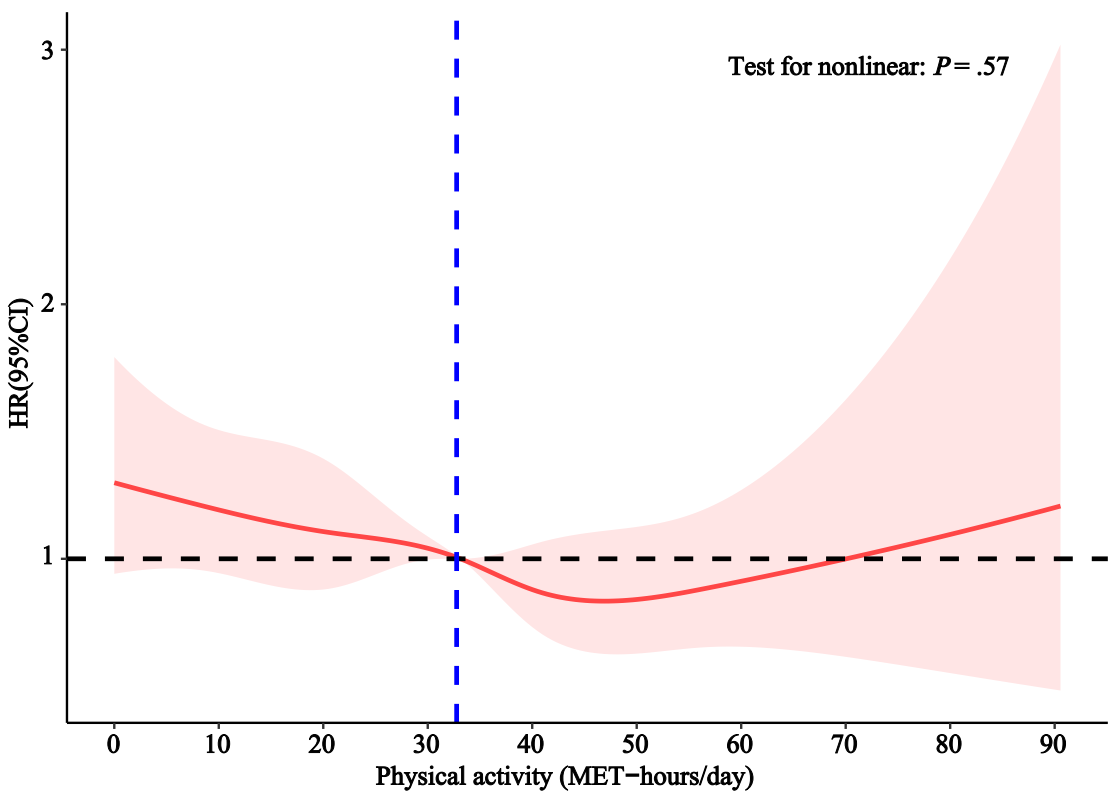


Figure S2. Restricted cubic spline plot for the association between physical activity and COPD among smoking men. Hazard ratios were adjusted for age, education level, household income, marital status, alcohol status, secondhand smoke exposure, meat consumption, fresh fruit consumption, sleep duration, body mass index, and household cooking fuel type.

Table S2. Association of physical activity with risk of incident COPD after additional adjustment or further exclusions

|  | Q1 | Q2 | Q3 | Q4 | *P* for trend |
| --- | --- | --- | --- | --- | --- |
|  | HR | HR^f^(95%CI) | HR(95%CI) | HR(95%CI) |  |
|  |  | Excluding first 1 year of follow-up | | |  |
| Total | 1 | 1.08 (0.94-1.24) | 0.89 (0.76-1.05) | 0.85 (0.70-1.02) | 0.03 |
| Men | 1 | 0.99 (0.82-1.21) | 0.85 (0.68-1.06) | 0.76 (0.59-0.99) | 0.03 |
| Women | 1 | 1.17 (0.96-1.43) | 0.94 (0.74-1.19) | 0.94 (0.71-1.22) | 0.42 |
|  |  | Excluding participants with poor self-rated general health | | |  |
| Total | 1 | 1.10 (0.96-1.26) | 0.93 (0.80-1.09) | 0.85 (0.71-1.02) | 0.052 |
| Men | 1 | 0.98 (0.82-1.19) | 0.87 (0.70-1.07) | 0.73 (0.56-0.94) | 0.01 |
| Women | 1 | 1.23 (1.01-1.51) | 1.00 (0.79-1.25) | 0.98 (0.75-1.27) | 0.64 |
|  |  | Additionally adjusted for sedentary leisure time | | |  |
| Total | 1 | 1.08 (0.94-1.23) | 0.94 (0.81-1.09) | 0.86 (0.72-1.03) | 0.08 |
| Men | 1 | 0.96 (0.80-1.16) | 0.88 (0.71-1.08) | 0.75 (0.58-0.96) | 0.02 |
| Women | 1 | 1.21 (1.01-1.47) | 1.01 (0.81-1.26) | 0.99 (0.77-1.27) | 0.74 |
|  |  | Additionally adjusted for occupation | | |  |
| Total | 1 | 1.08 (0.94-1.25) | 0.94 (0.81-1.10) | 0.87 (0.72-1.03) | 0.28 |
| Men | 1 | 0.95 (0.79-1.15) | 0.90 (0.73-1.12) | 0.79 (0.61-1.01) | 0.07 |
| Women | 1 | 1.22 (0.98-1.51) | 1.03 (0.81-1.32) | 1.08 (0.82-1.42) | 0.95 |

Hazard ratio in model was adjusted for age, sex, education level, household income, marital status, cigarettes consumption, alcohol consumption, secondhand smoke exposure, meat consumption, fresh fruit consumption, sleep duration, body mass index, and household cooking fuel type.

Table S3. Association of physical activity with risk of incident COPD by smoking status among men after additional adjustment for occupation

|  | Case, n | Incidence rate (per 1000 person-years), ‰ | Hazard ratios (95%CI) |
| --- | --- | --- | --- |
| Smokers |  |  |  |
| Q1 | 237 | 5.4 | 1 |
| Q2 | 201 | 4.6 | 0.90 (0.74-1.09) |
| Q3 | 147 | 2.5 | 0.85 (0.68-1.06) |
| Q4 | 92 | 1.7 | 0.75 (0.57-0.98) |
| *P* value for trend |  |  | 0.03 |
| Non-smokers |  |  |  |
| Q1 | 21 | 3.6 | 1 |
| Q2 | 22 | 4.8 | 1.66 (0.88-3.19) |
| Q3 | 16 | 3.1 | 1.69 (0.81-3.49) |
| Q4 | 8 | 1.9 | 1.16 (0.44-2.80) |
| *P* value for trend |  |  | 0.46 |

Hazard ratio: adjusted for age, education level, occupation, household income, marital status, alcohol status, secondhand smoke exposure, meat consumption, fresh fruit consumption, sleep duration, body mass index, household cooking fuel type.
